# The strategic biomarker roadmap for the validation of Alzheimer’s diagnostic biomarkers: methodological update

**DOI:** 10.1007/s00259-020-05120-2

**Published:** 2021-03-10

**Authors:** Marina Boccardi, Alessandra Dodich, Emiliano Albanese, Angèle Gayet-Ageron, Cristina Festari, Nicholas J. Ashton, Gérard N. Bischof, Konstantinos Chiotis, Antoine Leuzy, Emma E. Wolters, Martin A. Walter, Gil D. Rabinovici, Maria Carrillo, Alexander Drzezga, Oskar Hansson, Agneta Nordberg, Rik Ossenkoppele, Victor L. Villemagne, Bengt Winblad, Giovanni B. Frisoni, Valentina Garibotto

**Affiliations:** 1 German Center for Neurodegenerative Diseases DZNE-Standort Rostock/Greifswald, Gehlsheimer Str. 20, 18147 Rostock, Germany; 2 LANVIE - Laboratory of Neuroimaging of Aging, University of Geneva, Geneva, Switzerland; 3 Center for Neurocognitive Rehabilitation (CeRiN), CIMeC, University of Trento, Trento, Italy; 4 NIMTlab - Neuroimaging and Innovative Molecular Tracers Laboratory, University of Geneva, Geneva, Switzerland; 5 USI – Università della Svizzera Italiana, Institute of Public Health (IPH), Lugano, Switzerland; 6 Division of Clinical Epidemiology, Department of Health and Community Medicine, University of Geneva & University Hospitals of Geneva, Geneva, Switzerland; 7 LANE – Laboratory of Alzheimer’s Neuroimaging and Epidemiology, IRCCS Istituto Centro San Giovanni di Dio Fatebenefratelli, Brescia, Italy; 8 Wallenberg Centre for Molecular and Translational Medicine, University of Gothenburg, Gothenburg, Sweden; 9 Department of Psychiatry and Neurochemistry, Institute of Neuroscience & Physiology, The Sahlgrenska Academy at The University of Gothenburg, Molndal, Sweden; 10 NIHR Biomedical Research Centre for Mental Health and Biomedical Research Unit for Dementia at South London and Maudsley NHS Foundation, London, UK; 11 Institute of Psychiatry, Psychology & Neuroscience, King’s College London, London, UK; 12 Department of Nuclear Medicine, University Hospital Cologne, Cologne, Germany; 13 Division of Clinical Geriatrics, Center for Alzheimer Research, Department of Neurobiology, Care Sciences and Society, Karolinska Institutet, Stockholm, Sweden; 14 Theme Neurology, Karolinska University Hospital, Stockholm, Sweden; 15 Alzheimer Center Amsterdam, Department of Neurology, Amsterdam Neuroscience, Vrije Universiteit Amsterdam, Amsterdam UMC, Amsterdam, Netherlands; 16 Nuclear Medicine and Molecular Division, Geneva Medical Hospital, Geneva, Switzerland; 17 Departments of Neurology, Radiology & Biomedical Imaging, University of California, San Francisco, CA USA; 18 Alzheimer’s Association, Chicago, IL USA; 19 Faculty of Medicine, University of Cologne, Cologne, Germany; 20 German Center for Neurodegenerative Diseases (DZNE), Bonn/Cologne, Germany; 21 Molecular Organization of the Brain, Research Center Jülich, Institute of Neuroscience and Medicine (INM-2), Julich, Germany; 22 Clinical Memory Research Unit, Department of Clinical Sciences Malmö, Lund University, Lund, Sweden; 23 Memory Clinic, Skåne University Hospital, Malmo, Sweden; 24 Karolinska University Hospital, Theme Aging, Geriatric Clinic, Huddinge, Sweden; 25 Department of Clinical Memory Research, Lund University, Lund, Sweden; 26 Department of Molecular Imaging & Therapy, Austin Health, Melbourne, VIC Australia; 27 Department of Psychiatry, University of Pittsburgh, Pittsburgh, Pennsilvania USA; 28 Department of Neurobiology, Care Sciences and Society, Division of Neurogeriatrics, Karolinska Institutet, Stockholm, Sweden; 29 Memory Clinic, University Hospital, Geneva, Switzerland

**Keywords:** Biomarker, Alzheimer’s disease, Dementia, MCI, Mild cognitive impairment, Validation methodology

## Abstract

**Background:**

The 2017 Alzheimer’s disease (AD) Strategic Biomarker Roadmap (SBR) structured the validation of AD diagnostic biomarkers into 5 phases, systematically assessing analytical validity (Phases 1–2), clinical validity (Phases 3–4), and clinical utility (Phase 5) through primary and secondary Aims. This framework allows to map knowledge gaps and research priorities, accelerating the route towards clinical implementation. Within an initiative aimed to assess the development of biomarkers of tau pathology, we revised this methodology consistently with progress in AD research.

**Methods:**

We critically appraised the adequacy of the 2017 Biomarker Roadmap within current diagnostic frameworks, discussed updates at a workshop convening the Alzheimer’s Association and 8 leading AD biomarker research groups, and detailed the methods to allow consistent assessment of aims achievement for tau and other AD diagnostic biomarkers.

**Results:**

The 2020 update applies to all AD diagnostic biomarkers. In Phases 2–3, we admitted a greater variety of study designs (e.g., cross-sectional in addition to longitudinal) and reference standards (e.g., biomarker confirmation in addition to clinical progression) based on construct (in addition to criterion) validity. We structured a systematic data extraction to enable transparent and formal evidence assessment procedures. Finally, we have clarified issues that need to be addressed to generate data eligible to evidence-to-decision procedures.

**Discussion:**

This revision allows for more versatile and precise assessment of existing evidence, keeps up with theoretical developments, and helps clinical researchers in producing evidence suitable for evidence-to-decision procedures. Compliance with this methodology is essential to implement AD biomarkers efficiently in clinical research and diagnostics.

## Introduction

In 2014–2017, an international effort proposed the Strategic Biomarker Roadmap (SBR) as a methodological framework to improve the cost-effectiveness of the validation of Alzheimer’s disease (AD) (see Table 1 for a Glossary of terms used in this article) diagnostic biomarkers and facilitate regulators’ approval, refund, and implementation in daily practice [13]. This initiative consisted of adapting to the AD field, a methodological framework successfully used to validate diagnostic biomarkers in oncology [14], after adaptation from the methodology of drug development [15].

**Table 1:** Glossary detailing the meaning of the terms used in the 2020 Strategic Biomarker Roadmap

*Glossary*	
*Alzheimer’s disease*	We consider Alzheimer’s disease (AD) as the presence of extracellular amyloid-β plaques and aggregates of hyper-phosphorylated tau in neurofibrillary tangles, independently of the clinical expression of cognitive symptoms [1]
*AD dementia*	AD dementia denotes an acquired, insidious, and progressive cognitive and functional impairment due to AD, as defined in the National Institute of Neurological and Communicative Disorders and Stroke and the Alzheimer’s Disease and Related Disorders Association (NINCDS-ADRDA) criteria [2, 3]. However, a significant proportion (up to 30%) of AD dementia cases based on clinical criteria might have non-AD pathology [4–6]
*Analytical validity*	Ability of the assay (i.e., the ascertainment method used) to detect and/or quantify the biological or molecular target entity
*Assay*	Analytic procedure and methods measuring the presence, amount, or functional activity of a target entity
*Clinical stage*	The terms *dementia*, *MCI*, or *prodromal* denote the stage of clinical impairment and can relate to different pathophysiology
*Construct validity*	Degree to which a measure agrees with a theoretical construct. It entails the construct, i.e., the theory and current model of the target disorder, and the appropriateness of the inferential reasoning
*Criterion validity*	Extent to which a measure relates to other measures (concurrent validity). Criterion validity is tested against a gold standard
*Mild cognitive impairment (MCI)*	MCI refers to a population without, or with subtle, functional disability, but with an acquired objective cognitive impairment. Representing a clinical syndrome, it encompasses cases progressing to AD (about 50%) or non-AD dementia (about 10–15%) [7–10] as well as stable cases (about 35–40%). MCI cases positive to AD biomarkers can be defined as prodromal AD or MCI due to AD based on research diagnostic criteria [11, 12] and consistently also with the 2018 A/T/N framework [1]. The diagnosis of AD at the MCI stage represents the focus of the present review
*Non-AD neurodegenerative disease*	This term refers to all neurodegenerative disorders considered for the differential diagnosis, including a large pathological spectrum, e.g., hippocampal sclerosis, limbic-predominant age-related TDP-43, encephalopathy frontotemporal lobar degeneration, Lewy body disease, and multiple system atrophy
*Pathophysiology*	We use the terms AD and non-AD to denote the pathological presence or absence of amyloid-β plaques and aggregates of hyper-phosphorylated tau in neurofibrillary tangles
*Test*	Use of the assay to determine whether an individual is positive or negative to the target disease. It can be based on a continuous variable, with cutoffs used to define positivity, negativity, and gray zone

The SBR structures and the validation of AD diagnostic biomarkers into a systematic sequence of 5 phases each encompass primary and secondary aims. Phases 1–2 entail the assessment of analytical validity, Phases 3–4 clinical validity, and Phase 5 clinical utility. The framework specifies appropriate study designs, sample sizes, population, and gold- or admissible reference standards for each primary and secondary aim [14, 15]. Complying with the SBR logical sequence and methods increases the cost-effectiveness of validation studies by reducing errors of many kinds. For example, fulfilling all aims of analytical validity within Phase 2 allows to minimize the large amount of variability due to heterogeneous sampling procedures. Such variability was up to fivefold in the example of hippocampal segmentation with different protocols [16]. It cannot be amended *post hoc* and eventually results in the inability of the published data to enter formal evidence-to-decision (EtD) procedures and support evidence-based clinical or policy decision-making. An example for such failure is provided by the field of FDG-PET: the Cochrane review analyzing the extensive literature on its diagnostic accuracy in detecting AD in MCI patients found exceedingly large variability of results and concluded that no clinical recommendation could be issued based on such data [17]. Appraising the validation status of diagnostic biomarkers based on the SBR framework helps to map the validation steps that are properly completed, those in need of further confirmation, and the gaps requiring urgent investigation, before proceeding and collecting additional data that would otherwise lack validity, being based on faulty premises. Thus, complying with this methodology helps generating data eligible to formal EtD procedures [18], i.e., objective and transparent decision-making procedures for clinical or policy contexts, that can be based on available literature transparently and directly, with minimum intervention by expert panels. EtD ineligibility leads, on the other side, to the need of consensus by experts, who can only make decisions based on individual expertise and on the available, but faulted, data.

In 2017, we used this framework to assess the validation status of the neuropsychological assessment (viz., episodic memory test) as a gateway to biomarker-based diagnosis [19], and of most consolidated AD biomarkers at that time, i.e., amyloid imaging [20], CSF [21], hippocampal atrophy [22], FDG-PET [23], and biomarkers for dementia with Lewy bodies [24], based on evidence published until 2015. In the present work, we revised the SBR to update it to the current A/T/N framework for research on AD and related disorders [1] and to enable proper assessment of biomarkers of tau pathology [25–29]. Such update was required, in that the diagnostic criteria adopted in 2017 entailed a relatively unclear role of Tau in the diagnostic procedure of AD. Instead, the new A/T/N framework [1] (a) requires tau positivity to formulate a diagnosis of *clinical* AD (AD dementia, or MCI due to AD, as opposed to *Alzheimer’s pathologic change* defined by biomarkers), and (b) depicts cases with positive tau and negative Aβ as belonging to a non-AD, but still to a dementing neurodegenerative disorders continuum that is relevant to the clinical aim of providing patients with accurate and timely diagnosis. These features can impact the kind of required or admissible gold/reference standards and the design of validation studies, as well as the exact meaning of positive tau biomarkers in the diagnostic procedure, thus requiring to check and possibly revise some aspects of the 2017 SBR.

## Methods

This work is part of a wider initiative, aimed to use the SBR and assess the validation status of biomarkers of tau pathology [25–29]. The initiative consisted of a workshop held in Geneva, November 11–12, 2019. The PI (Valentina Garibotto) convened the Alzheimer’s Association and 8 expert groups on AD biomarker research, namely, those led by Giovanni Frisoni, Alexander Drzezga, Oskar Hansson, Agneta Nordberg, Rik Ossenkoppele, Gil D. Rabinovici, Victor L. Villemagne, and Bengt Winblad. Within this wider initiative, this work aimed to define the methodology allowing some of these expert groups to assess the validation status of AD biomarkers consistently, in line with the theoretical development of the AD field. To provide such updated methodology, we discussed the adequacy of the 2017 SBR methods specified for each phase’s primary and secondary aim with dedicated methodologists (EA, AGA). We dedicated specific attention to the features of the biomarkers of tauopathy, which were just being developed during the previous SBR initiative (2014–2017) and to the new AD research diagnostic criteria [1]. We have then formulated an updated proposal, which was presented and discussed at the Geneva workshop. The general discussion contributed to clarify the issues entailed in the current scenario of AD biomarker development, fix required updates, and outline needs for future developments. Four of the participant expert groups (viz., those led by Agneta Nordberg, Oskar Hansson, Rik Ossenkoppele, and Alexander Drzezga) have then assessed the validation status of CSF-, plasma- and imaging-tau AD biomarkers based on the evidence published until July 2020. Research strings and specific methods are reported in the five dedicated reviews [25–29].

In addition to the revision of the SBR methodology, we have also provided updates on the assessment of aim achievement within the dedicated reviews. Such updates were defined based on preliminary data on the validation status of biomarkers of tauopathy and on other methodological considerations, in particular the fact that the same groups involved in biomarker development were assessing the available studies.

## Results

Most of the methods used in 2017 were still appropriate to assess biomarker of tauopathy and other biomarkers within the A/T/N framework. The aspects that required adjustment included the definition of the study design eligible to assess aim achievement, the admitted reference standards for Phases 2–4, the formal data extraction for the evidence assessment, and edits to the definition of aim achievement. Below we detail these aspects along with the other key features of the initiative consistent with 2017.

### Context of use (purpose, population, and nature of disease)

As in 2017, the context of use remains the diagnosis in people referred or autonomously referring to memory clinics for cognitive complaints. The aim of this context of use consists of using biomarkers to detect AD and, primarily, identify people whose impairment is likely to progress to a dementia stage; consistently, we use “progression at follow-up” as a reference standard for the biomarker. However, we underline that despite this perspective and methods, the aim of the SBR is to validate *diagnostic*, and not *prognostic* biomarkers. Consistent with 2017, only patients with objective impairment at formal cognitive assessment would access the full diagnostic workup, and we focus on the MCI stage.

### Context of use: 2020 update

Relative to the target disorder, we keep referring primarily to AD, namely, to the neurodegenerative dementing disorder characterized by brain amyloidosis and tauopathy, consistent with the 2018 A/T/N framework [1]. In this case, the difference from the 2017 framework regards the theoretical research construct behind the definition of AD as a pathophysiological disorder, although it does not affect the clinical picture of the target disorder, nor the formal gold standards, that should ideally entail both clinical progression at follow-up examination and confirmation at pathology. However, within the A/T/N framework, the tau biomarkers can also detect patients with non-AD neurodegenerative disorders. This option is therefore now expressly included by our initiative. It is consistent with the use of clinical progression as a reference standard and with the ultimate aim of detecting patients with dementing neurodegenerative disorders in clinical practice.

### Analytical validity

(See Table 1 for a Glossary of terms) The primary and secondary aims of Phases 1–2 studies are the same as in the 2017 assessment (Table 2). Briefly, analytical validity relates to the ability of the assay, that may be later used as a diagnostic test, to detect the alteration of interest. Thus, the gold standard for Phases 1–2 studies is pathology, and the primary and secondary aims are set to determine the features of the assay in order to ascertain its potential usefulness as a diagnostic test and to specify standard operating procedures to guarantee informative and reliable measurements within and across laboratories.

**Table 2:** Phases for the formal validation of diagnostic biomarkers according to the Strategic Biomarker Roadmap (SBR) for case finding in oncology [15], and for the diagnosis of Alzheimer’s disease in patients with MCI, according to the 2017 adaptation from oncology [13, 14] and the 2020 update

	Phase			Aim				Comments	
	Oncology	SBR 2017 AD (2011 criteria)	SBR 2020 AD (stage within A/T/N framework)	Oncology		SBR 2017 AD (2011 criteria)	SBR 2020 AD (stage within A/T/N framework)	SBR 2017	SBR 2020
Analytical validity (target: assay)	Phase 1			Context of use: Purpose: screening and case finding Population: preclinical/at risk		Context of use: Purpose: diagnosis Population: patients referred/-ing to clinical centers due to cognitive complaints		Diagnostic criteria for Alzheimer’s disease are based on clinical features and include the use of biomarkers to increase the certainty on the etiological diagnosis of AD	Biomarker profiles combining amyloid, tauopathy, and neurodegeneration lie along an AD or non-AD continuum
	Preclinical exploratory studies			*PA*	To identify and prioritize leads for potentially useful biomarkers				
	Phase 2				Phase 2 assesses analytical validity, i.e., the ability of the assay to detect the alteration of interest				
	Clinical assay development for clinical disease			*PA*	To estimate the true and false positive rate or ROC curve and assess its ability to distinguish subjects with and without the disease			*Design*: case-control. *Population*: pathological specimens from any AD stage. *Gold standard*: pathology. *Outcome*: case-control separation (sensitivity, specificity, true positive ratio, false positive ratio, and ROC curves)	*Reference standard* (clinical progression at follow-up; positivity to another AD biomarker) is allowed due to limited accessibility to pathology. However, this is a key limitation in studies of analytical validity. Thus, the use of a reference standard in Phase 2 studies should be accepted with warning (*)
				*SA 1*	To optimize procedures for performing the assay and to assess its reproducibility within/between laboratories			The lack of reliable standard procedures to perform the essay implies that subsequent studies and diagnostic procedures include uncontrolled variability that cannot be amended post hoc. This variability hampers the eligibility of published data for evidence-to-decision procedures	
				*SA 2*	To determine the relationship between biomarker measurements made on tumor/brain tissue and the biomarker measurements made on the noninvasive clinical specimen			Gold standard is pathology	*Reference standard* (clinical progression at follow-up; other biomarker) is admitted due to limited accessibility to pathology. However, this is a key limitation in studies of analytical validity ➔ accept with warning (*)
				*SA 3*	To assess factors (e.g., sex, age, etc.), associated with biomarker status or level in control subjects			If such factors affect the biomarker, subpopulations need different thresholds for positivity	
				*SA 4*	To assess factors associated with biomarker status or level in diseased subjects—in particular, disease characteristics such as stage, histology, grade and prognosis	To assess factors associated with biomarker status or level in cognitively impaired subjects—in particular, disease characteristics such as stage, histology, grade, and prognosis			
Clinical validity (target: test performance)	Phase 3				Clinical validity assesses the performance of the assay, developed in Phase 2, now used as a diagnostic test. Phase 3 studies are performed in well controlled experimental settings, examining cohorts from research centers or academic memory clinics; the biomarker is assessed, but not used to formulate the clinical diagnosis for patients *Outcome*: case-control separation (sensitivity, specificity, true positive ratio, false positive ratio, and ROC curves)				
	*Retrospective* longitudinal repository studies	*Prospective* longitudinal repository studies	*Longitudinal* repository studies	*PA 1*	To evaluate, as a function of time *before clinical diagnosis*, the capacity of the biomarker to detect *preclinical disease*	To evaluate, as a function of time *in the prodromal stage (MCI)*, the capacity of the biomarker to *predict conversion to AD dementia*		*Design*: observational prospective longitudinal case-control studies (in the absence of nested case-control longitudinal cohorts allowing retrospective examination) *Population*: MCI *Gold standard*: pathology *Reference standard*: progression to dementia at 3 years (if pathology is lacking) *Outcome*: case-control separation	*Design*: in addition to the 2017 designs, cross-sectional studies, containing known risk factors for dementia (e.g., confirmed brain amyloidosis, APOE-e4, etc.) are admitted as contributing construct validity evidence. Moreover, repositories may now allow *retrospective* studies also for biomarkers for the diagnosis of neurocognitive disorders *Gold standard*: clinical progression should not be considered just an acceptable reference standard in the lack of pathology but should be included in the definition of the gold standard (clinical progression + pathology). *Reference standard*: consistently with the considerations on cross-sectional design, biomarker characterization or other features can contribute to construct validity. However, clinical progression generates stronger evidence than other reference standards based on construct validity. *Outcome*: case-control separation (sensitivity, specificity, true positive ratio, false positive ratio, and ROC curves)
				*PA 2*	To define criteria for a positive screening test in preparation for Phase 4	Define criteria for a positive diagnostic test for MCI due to AD, in preparation of Phase 4		“in preparation of Phase-4”: in phase 3, the biomarker is assessed in strictly experimental conditions. Patients are tested, but their diagnosis is NOT based on the biomarker under examination. Here, the biomarker’s features are adjusted to allow its use for clinical diagnosis in Phase 4	
				*SA 1*	To explore the impact of covariates on the discriminatory abilities of the biomarker before clinical diagnosis			Note the difference between Phase 3_SA1 and Phase 2_SA3–4: in Phase 2, covariates are assessed relative to their effect on status or level of the biomarker and on the threshold for positivity, in order to define *the assay*. In Phase 3, covariates are explored relative to their effect on the discriminatory ability of the biomarker, i.e., in using the *assay as a test*	
				*SA 2*	To compare markers to select the most promising			SA2: Compare markers—study design must quantify not only the diagnostic accuracy of the target biomarker (index test) but also the accuracy of the traditional or alternative procedure, in order to quantify the *incremental* diagnostic value of the target biomarker. The compared *outcomes are* case-control separation (sensitivity, specificity, true positive ratio, false positive ratio, and ROC curves)	
				*SA 3*	To develop algorithms for screening based on combinations of markers	Develop algorithms for the biomarker-based diagnosis of MCI in preparation of Phase 4		Phase 3_SA2–3 provide the data to combine markers and define diagnostic algorithms guiding clinicians’ use of biomarkers in Phase 4. *Outcome*: case-control separation (sensitivity, specificity, true positive ratio, false positive ratio, and ROC curves) obtained with combinations of biomarkers	
				*SA 4*	To determine a screening interval for Phase 4 if repeated testing is of interest	To determine an interval able to detect a meaningful change of biomarker status or level in progressing MCI			
	Phase 4				In Phase 4, the biomarker, still under investigation, is used also in non-academic clinical contexts to support patient diagnosis. The experimental use of the biomarker should be made explicit to patients. Phase 4 provides validation data on the use of the biomarker in real-world rather than strictly controlled conditions. Sample sizes are larger than in Phase 3				
	Prospective screening studies	Prospective diagnostic studies		*PA*	To determine the operating characteristics of the biomarker-based screening test in a relevant population by determining the detection rate and the false referral rate	To determine the operating characteristics of the biomarker-based diagnostic test in MCI patients in the memory clinics population		*Design*: prospective longitudinal studies (studies at this stage involve testing people and lead to diagnosis and treatment). *Population*: same as Phase 3 (size: hundreds, from tertiary referral centers). *Gold/reference standard*: like Phase 3 *Outcome*: proportion of cases correctly diagnosed; baseline correlates of biomarker positivity (age, severity, etc.); compliance with the program; disease-associated morbidity, quality of life, costs	Design, population, gold standard: same as Phase 3 Outcome: same as 2017
				*SA 1*	To describe the characteristics of tumors detected by the screening test—in particular, with regard to the potential benefit incurred by early detection	To describe the characteristics of the neurodegenerative disorder detected by the diagnostic biomarker—in particular, with regard to the potential benefit incurred by early detection			
				*SA 2*	To assess the practical feasibility of implementing the screening program and compliance of test-positive subjects with workup and treatment recommendations	To assess the practical feasibility of implementing the biomarker-based diagnostic procedure and compliance of test-positive subjects with workup recommendations.			The emotional and social implications related to positive test results within the diagnostic procedure may need to be assessed and taken into account, to increase compliance to workup recommendations. This was done in oncology, also based on a counseling and patient involvement that is not yet fully developed in the field of dementia
				*SA 3*	To make preliminary assessments of the effects of screening on costs and mortality associated with cancer	To make preliminary assessments of the effects of biomarker-based diagnosis on costs and burden associated with AD			Outcome definition is still insufficient to cover this aim properly [30] and should be solved with priority
				*SA 4*	To monitor tumors occurring clinically but not detected by the screening protocol	To monitor AD dementia/neurocognitive disorders occurring clinically but not detected by the biomarker-based diagnostic procedure			
Clinical utility	Phase 5				Phase 5 entails surveillance studies on thousands of subjects				
	Cancer control studies	Disease control studies		*PA*	To estimate the reductions in cancer mortality afforded by the screening test	To estimate the effects of biomarker-based diagnosis on disease-associated mortality, morbidity, and disability		Design: surveillance system of accepted practice *Population*: = same as Phase 4 (size: thousands, from secondary referral centers) *Outcome*: proportion of cases correctly diagnosed; baseline correlates of biomarker positivity (age, severity, etc.); compliance with the program; disease-associated morbidity, quality of life, costs	
				*SA 1*	To obtain information about the costs of screening and treatment and the cost per life saved	To obtain information about the costs of biomarker-based diagnosis per quality-adjusted years of life			
				*SA2*	To evaluate compliance with screening and workup in a diverse range of settings.	To evaluate compliance with biomarker-based diagnostic workup in a diverse range of settings.			
				*SA 3*	To compare different screening protocols and/or to compare different approaches to treating screen-detected subjects in regard to effects on mortality and costs	To compare different biomarker-based diagnostic protocols and/or to compare different approaches to treating biomarker-based diagnosed subjects in regard to effects on quality-adjusted years of life, mortality, and costs			

*AD* Alzheimer’s disease, *MCI* mild cognitive impairment, *PA* primary aim, *SA* secondary aim. * In AD, tissue is not accessible as in other contexts, like oncology. We assessed Aims completion weighing such feasibility issues. However, especially in the Analytical Validity stage, aim achievement should be assessed in studies using pathology as the gold standard (see Table). “Achievement” without pathology should be considered "with warning" to underline such intrinsic limitation, and the need of producing or replicating this kind of evidence when feasible.

### Analytical validity: 2020 update

**Fig. 1:**
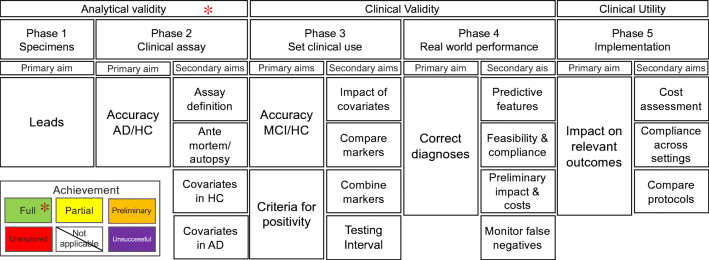
Flowchart denoting the sequence of the primary and secondary aims of the 5 validation phases of the Strategic Biomarker Roadmap (SBR), as updated from the 2017 framework [13, 14]. The achievement color codes denote the possible outcomes for the assessment of the validation status of biomarkers based on the 2020 SBR (Green: *Fully achieved*, when available scientific evidence was replicated in at least two independent well-designed and adequately powered samples in studies. Yellow: *Partly achieved*, when available evidence needs further replication in studies with better methodology or greater power. Orange: *Preliminary evidence*, when only preliminary data are available from ongoing projects, or published evidence is limited or inconsistent. Red: *Not achieved*, when no evidence is available, or studies are known to be ongoing or to have generated data at the time of the assessment. White: *Not applicable*, when the aim is not pertinent to the biomarker under consideration. Purple: *Unsuccessful*, when evidence is available, demonstrating that the biomarker failed the validation step). Aim achievement should ideally be assessed by raters independent from those involved in the assessed studies. Moreover, the assessment should be based on formal procedures thoroughly examining risks of bias and other methodological parameters, more exhaustively reported in the supplemental tables available at https://drive.switch.ch/index.php/s/4reUTSuqNZHyIC8). In this initiative, the young researchers from the expert groups used this structure to perform data extraction and facilitate sounder evidence assessment by independent methodologists (*see legend in Table 2)

In this update, we admitted that some Phase 2 studies could have only progression at follow-up, or other reference standard like positivity to a different AD biomarker, as an acceptable reference standard, due to the fact that brain histology is usually not accessible at the time of the biomarker assessment. However, this should be considered with warning of methodological fault for Phase 2 studies, and producing evidence-based on proper gold standard should anyway be considered a research priority (see asterisk in Fig. 1 and Table 2).

### Clinical validity

Clinical validity, assessed in Phases 3–4 studies, is aimed to use the assay defined in Phases 1–2 as a test to detect the target disease and to assess its diagnostic performance (sensitivity, specificity, likelihood ratios, and area under the curve). The primary and secondary aims of Phase 3 are intended to adjust the test features and its use in the diagnostic procedure, e.g., to define thresholds by accounting for covariates as assessed in Phase 2 and to achieve the desired discrimination accuracy. In order to tune the diagnostic test based on the data from the essay, covariates, the desired discrimination accuracy, and the other basic properties, all studies in Phase 3 are performed in strictly controlled experimental conditions. Thus, even when patients come from memory clinics, the biomarker itself is not used to formulate their clinical diagnosis, but is only collected for research purposes. The biomarker’s features are thus assessed and adjusted, to prepare its use in Phase 4 studies. In Phase 4, the biomarker is tested in real clinical contexts where it is also used to support clinical diagnosis for patients, although within a research framework. Therefore, the information collected in Phase 4 studies informs about the diagnostic performance of the biomarker in real-world contexts, where patients are not strictly selected and may have a variety of comorbidities, diagnostic protocols are clearly defined but may not be systematically followed across centers, etc. In Phase 4, also non-academic memory clinics use the biomarker and contribute data for its validation. Usually, sample sizes are larger (hundreds) than those in Phase 3 (dozens).

All the primary and secondary aims for clinical validity are fully consistent with the 2017 framework.

### Clinical validity: 2020 update

Similar to the original framework proposed in 2001 for oncology, repositories of pertinent biological samples are now available and allow to perform retrospective longitudinal studies that were not possible in 2017 (the 2017 SBR only included “prospective” longitudinal studies). Moreover, it is clear at this time that a structured plan to define clinically meaningful outcomes is urgently needed to set the bases for proper Phase 5 studies (see the “Clinical utility” section; [30]).

### Clinical utility

In Phase 5, practical issues like implementability, health benefit, and cost-effectiveness are assessed specifically and systematically, leveraging the preliminary information collected in Phase 4. All primary and secondary aims of clinical utility are consistent with the 2017 framework.

### Clinical utility: 2020 update

The outcomes to be considered in the assessment of cost-effectiveness not only differ from those of oncology but are still scarcely defined for the field of AD itself [30]. The definition of outcomes should be relevant for end-users [31] and clinically meaningful [32]. Consensual definitions of clinical meaningfulness involving end-users are still lacking and should be formulated with priority, possibly within Phase 4 studies. Only studies assessing biomarker performance relative to clinically significant and patient-relevant endpoints can produce data that can enter evidence-to-decision (EtD) procedures for clinical and policy decision-making [31].

### Gold standard

Pathology is the required gold standard for Phases 1–2 assessing analytical validity. Ideally, pathological confirmation should be obtained at all validation steps in all phases. However, clinical progression at follow-up is used as an admissible reference standard for clinical validity studies (Phases 3–4).

### Gold standard: 2020 update

The inaccessibility of brain tissue led to admit reference standards as acceptable, despite warning, even in Phase 2 studies for different reasons. First, concrete hurdles hamper the performance of autopsy studies. Second, even when autopsy is performed, tissue examination may be too a long time apart from biomarker assessment in the relevant clinical phase (e.g., MCI), with weak connection between the two kinds of data. Third, at present, the ultimate practical clinical interest focuses more on identifying persons with cognitive impairment who will probably *progress to dementia*, than on identifying the exact underlying pathology. Finally, we are developing an increasing awareness of the complexity of AD and related disorders [33], and, relative to clinical diagnosis, “progression to dementia” is considered by some as an even more appropriate gold standard than autopsy. This is even more true in the case of tau biomarkers: indeed, the A/T/N criteria not only require positivity to tau pathology to define clinical AD but also categorize individuals with positive tau and negative Aβ as belonging to the continuum of non-AD progressive neurodegenerative disorders (Table 2 in [1]). Since the context of use defined for the SBR consists of diagnosing people with MCI in memory clinics, this potential use of tau biomarkers should not be ruled out for not serving an AD diagnosis specifically. This set of reasons led to admit less stringent reference criteria as a mandatory methodological decision. We will use the term “reference standard” for the sake of methodological rigor, to indicate the lower level of evidence provided by the lack of confirmation of AD (or of other non-AD neurodegenerative disorders) at pathology, although some of the above reasons may support the use of clinical progression as no less appropriate for some goals. Relative to the use of clinical progression as a reference standard, this was already admitted for clinical validity studies (Phases 3–4) in 2017. The 2020 update formally included the possibility to admit clinical progression as a reference standard also for Phase 2 (very few studies with pathology could be performed for tau imaging to date) [34]. With progressing validation of AD biomarkers, positivity at an alternative AD biomarker can be used as a reference standard contributing construct validity, although this should be done only within the aims that are not intended to compare or combine different biomarkers. The evidence obtained using positivity to an alternative AD biomarker alone (or to other features, like APOE) is weaker than that provided by studies using clinical progression as reference standard. One reason is that the other biomarkers are still under investigation themselves, and without complete formal validation, they cannot become the new proper gold or reference standards. Moreover, the evidence so collected cannot be directly associated to a *progressive* neurodegenerative condition and, relatively to tau biomarkers, cannot account for non-AD neurodegenerative disorders. However, such studies can be taken into account as providing evidence of construct validity specifically to AD. Admitting such reference standard affects the study design to be considered: cross-sectional studies thus are eligible with the 2020 SBR, in addition to longitudinal studies.

### Methods to perform the biomarker-specific reviews

As in 2017, we asked young researchers from the involved expert groups to identify the clinical studies addressing the ABR aims, possibly on multiple databases (e.g., PubMed, Embase, and Cochrane; reviews were not included but used to identify additional original article). Inclusion criteria for papers’ selection were as follows: (1) Manuscript type: full manuscript; (2) Population of interest: The target population was AD diagnosed according to validated clinical diagnostic criteria as defined in the Glossary; (3) Language and time span: only papers published in English and up to July 2020. Relevant previous literature from personal knowledge and tracked from reviews was included. Book chapters, conference abstract, and case reports were excluded.

### Methods to perform the biomarker-specific reviews: 2020 update

In order to make reviews more systematic than those performed in 2017, for each review we asked that an independent researcher replicate the literature search for random aims, to ascertain the replicability of findings, and check the data extraction accuracy. Based on the output, search strings as well as paper selection were updated to improve both searches and their replicability.

### Assessment of aim achievement

The fulfillment of each aim was assessed examining the available data with the same criterion used in the 2017 SBR (Fig. 1 and Legend to Fig. 1).

### Assessment of aim achievement: 2020 update

In addition to the 2017 assessment, we included the possibility of coding possible failures along the validation course (purple box in Fig. 1): namely*,* an aim can be defined *Unsuccessful*, when evidence is available, demonstrating that the biomarker failed the validation step. Although our method to assess aim achievement (See Fig. 1 legend) does address somehow the adequacy of studies’ methodology, this is weak and liable to bias. Therefore, in addition to this reference assessment criteria, we have provided tables structuring data extraction and reporting based on methodological guidance for formal evidence assessment [18, 35, 36] and on previous adaptations to diagnostic biomarkers for AD [37] (see Supplemental Material at https://drive.switch.ch/index.php/s/4reUTSuqNZHyIC8). Indeed*,* proper evidence assessment requires specific analyses [18, 35, 36] thoroughly assessing risks of bias and other parameters possibly increasing (e.g., strong effect) or decreasing (e.g., large confidence intervals) the quality of the produced evidence. While such assessment goes beyond the aim of the current initiative, we nonetheless deemed relevant to set the bases and facilitate a future development towards such formal evidence assessment. Thus, we have provided tables for data extraction and we asked the researchers performing the reviews to fill them and report the main features that allow for better understanding and assessment of potential biases (e.g., limited sample size, lack of matched controls, case-control in place of cohort design study, reference standard in place of gold standard, lack of blinding in the assessment of biomarkers, short follow-up duration or excessive number of drop outs, etc.) [18, 36]. These tables, adapted from previous evidence assessment for diagnostic biomarkers for neurocognitive disorders [37], are filled with the data extracted from the studies included in the reviews— [25–29]—and made available to the readers to complement our overall assessment (https://drive.switch.ch/index.php/s/4reUTSuqNZHyIC8).

## Discussion

In this work, we have revised the 2017 Biomarker Roadmap methodology [13, 14] to allow for the assessment of biomarkers of tauopathy, as well as that of the other diagnostic biomarkers of AD and related disorders, consistently with the 2018 A/T/N research framework. Most of the 2017 methodology remains valid (the Biomarker Roadmap was first launched late in 2014); aligning to the A/T/N framework [1] did not significantly affect the context of use (besides a more explicit inclusion of non-AD conditions), since the validation of diagnostic biomarkers is currently aimed to a clinical use and relies necessarily on a clinical definition of the target disorder. However, we have admitted a larger variety of reference standards based on construct validity, consistent with increasingly acknowledged complexity [33] and an atheoretical approach. Also, under the light of recent studies, we have clarified some methodological issues to provide guidance in applying the SBR while performing new research or assessing available validation studies of diagnostic biomarkers for AD. Finally, we have outlined research priorities for the next validation studies (e.g., the need to define clinical outcomes to assess societal impact in Phase 5). This work leverages previous efforts, namely, in oncology [15] and on the 2017 SBR [13, 14], and tries to capitalize on requirements for ultimate implementation of biomarkers in clinics based on the methodological constraints for regulatory purposes [18, 31, 35, 36].

### Role of tau biomarkers

The A/T/N criteria define a clear role of tau biomarkers in the diagnostic procedure of patients complaining about cognition. In particular, (a) their positivity is required to define *clinical* AD, and (b) their positivity in Aβ-negative patients denotes the presence of a neurodegenerative disorders belonging to a non-AD continuum. These are the main features underlying the need for a revision of the SBR. The informative value of biomarkers of tauopathy for either AD or non-AD neurodegenerative disorders is helpful for clinical use; therefore, this methodology is generically aimed at detecting AD, but is not meant to exclude other dementing neurodegenerative disorders.

### W*ider set of reference standards admissible to assess aim achievement*

The limited accessibility to brain histology hurdles the availability of the gold standard for AD biomarkers; limitations in following up patients, as well as inconsistencies in the assessment of progression across clinics, limit the validity and reliability of the detection of conversion to dementia and of its use as a satisfactory reference standard. However, our evolving construct of AD and the progressing validation of other AD biomarkers allow to consider additional reference standards (e.g., positivity to other AD biomarkers, to APOE-ε4, etc.). These can support the validation of AD biomarkers through cross-sectional studies. The downside of this approach is that such studies produce evidence with heterogeneous levels of strength, which needs therefore to be weighted when assessing the achievement of the validation aims.

### Evidence assessment: a mandatory step

When it comes to evidence-based decisions, published evidence must be examined based on formal evidence assessment [36]. This is originally thought to serve clinical and policy decision-making. However, this relates also to our effort of assessing aim achievement based on the SBR. In this initiative, we have still assessed aim achievement based on the 2017 criteria (Legend to Fig. 1) for the sake of feasibility. However, this approach is limited for different reasons. First, the criteria for achievement disregard a formal evidence assessment of all possible risks of bias; second, the introduction of different kinds of reference standards requires to weight the different strength of the produced evidence; third, this whole assessment should be done by methodologists with specific background, and not involved in the validation of the assessed biomarkers themselves. Despite the current limitations, we have structured a detailed data extraction, adapted to our specific context as recommended by evidence assessment approaches like GRADE [35, 36] and QUADAS [38] (https://drive.switch.ch/index.php/s/4reUTSuqNZHyIC8) as a step forward, and invited the researchers tasked with the review search to fill the data extraction files. This should enable the readers, independent methodologists, or future initiatives, to perform a formal assessment of the strength of the evidence of current data, and may serve next developments of the SBR.

A methodological resource in support of both reporting and study design may be provided by promoting compliance to the available reporting guidelines (https://www.equator-network.org; https://www.equator-network.org/?post_type=eq_guidelines&eq_guidelines_study_design=diagnostic-prognostic-studies&eq_guidelines_clinical_specialty=0&eq_guidelines_report_section=0&s=). Methodological research priorities may include ascertaining whether these guidelines exhaustively reflect SBR aims specific to biomarkers for dementing neurodegenerative disorders, and either adapt them, or, if already adequate, promote their use in the field. In this initiative, we have complied with reporting guidelines for the reviews performed to assess the specific biomarkers [39]. Then, the tables for data extraction may be refined consistently. Meanwhile, our tables can provide, besides the assistance to evidence assessment, also an interim guidance to structure the reporting of the information specifically required to produce data eligible to EtD [18, 31, 35, 36].

### Research priorities for the methodological development of the SBR

Although the steps of the SBR were defined in a rather systematic way, key components are still missing and should be considered methodological priorities. For example, there is no agreement on the definition, selection, and assessment of the outcomes allowing reliable assessment of the impact of diagnostic biomarkers, meant to assess clinical utility in Phases 4–5 [30]. The currently used measures of biomarkers’ diagnostic accuracy or physicians’ diagnostic confidence are only indirectly connected with the clinically relevant and patient-relevant outcomes required by regulators [31, 32, 35, 40], and these are in turn far from being consensually agreed upon. Defining such outcomes is particularly urgent, since most AD biomarkers are already being validated in Phase 4 studies. Relative to Phase 4, we also underline that in this phase, biomarkers are not only evaluated in real-world clinical contexts but are also used to support patients’ diagnosis in such contexts. The still experimental use of such biomarkers should be made explicit to patients [41], and protocols for proper communication of the concept of risk, and of diagnosis itself, should be developed. As for the research methodology [15], other medical fields may also in this case serve as a guide (e.g., genetics for the communication of risk; oncology for the communication of diagnosis and counseling).

### Ultimate benefit of complying with the SBR

Complying with the SBR implies following the validation steps in the outlined order and with the study designs and kinds of variables detailed in Table 2. Lack of compliance with any of these characteristics leads to include variability and gaps that cannot be amended post hoc and that ultimately determine the ineligibility of available data to evidence-based procedures for decision-making [17, 37]. Complying with the SBR validation steps, sequence, and methodology is therefore necessary to collect proper evidence having sufficiently high quality to support the downstream implementation of biomarkers into practice. The reviews assessing the validation status of biomarkers and mapping gaps and research priorities— [25–29]—are meant to detect gaps early on in the validation procedure and inform on how to adjust it, before extensive efforts are deployed in downstream studies that would provide faulty or biased data. Thus, keeping the methodology updated and performing periodical assessments of validation proceedings can improve the cost-effectiveness of biomarker validation.

### Limitations

The major limitation of the whole initiative consists of the difficulty to access brain tissue and the rare availability of pathology data providing the gold standard. This limitation is particularly important for the phase of analytical validity. To overcome this hurdle and proceed to the validation of diagnostic biomarkers for dementing neurodegenerative disorders, we have admitted positivity to other biomarkers in virtue of construct validity. However, the use of other biomarkers is not always possible (e.g., when different biomarkers are themselves object of comparison, e.g., in Phase 3, secondary aims 2 and 3). Moreover, such use is anyway subject to potential circularity: all biomarkers are still under investigation, and they would provide a reference standard for a specific disease (e.g., AD rather than other disorders that do not lie on the AD continuum, but still have tau positivity) based on our current (but not necessarily final) construct of AD. The alternative solution of clinical progression is apparently weaker, but actually sensible. Indeed, the ultimate clinical target consists of detecting progressive neurodegenerative disorders. Especially for biomarkers of tauopathy, clinical progression properly includes non-AD neurodegenerative disorders, consistently with the A/T/N criteria. However, in this case the limitation consists in possible heterogeneous measurements of progression across studies, weakening its validity and reliability and hampering comparability. Relative to the methods for performing the reviews, besides the mentioned limitations consisting of the lack of formal evidence assessment performed by independent methodologists, we also underline that the SBR effort has a relatively limited power to synchronize validation studies performed by independent groups. Working independently and on heterogeneous datasets, biomarker validation cannot proceed in a perfectly systematic way. Availability of datasets containing information for Phase 4 studies, for example, leads to initiate such studies despite incomplete evidence from previous phases. This problem is not unique to the AD field; however, measures may be taken to limit such mismatched proceeding and help integrate gaps in support of the whole process (see, e.g., the production of recommendations [42, 43], to support clinicians in a most rationale [44] use of such biomarkers in the lack of evidence for their combined use: such recommendations increase the reliability of clinical procedures as well as the consistency of the data that are collected in clinics and also used for research purposes). Finally, the A/T/N criteria are meant for research, and not for clinical diagnostic use; however, the whole theoretical and research effort on diagnostic biomarkers aims to the ultimate aim of improving clinical procedures for patients. Using the most advanced theories available to improve the methodology for validating biomarkers, while keeping into account the final concrete goal, can lead to apparent inconsistencies, for example, the validation of tau biomarkers for supporting AD diagnoses, but also the diagnosis of non-AD neurodegenerative disorders.

## Conclusions

The field of AD diagnostic biomarkers is progressively approaching that of oncological biomarkers [15]. With the A/T/N framework, biomarkers are examined and assessed for their individual contribution to an AD or non-AD profiles, allowing more precise diagnosis that is more independent on specific theories on AD pathogenesis; the availability of sample repositories now allows studies with retrospective design in addition to the only prospective studies of MCI clinical trajectory; and most recent biomarkers like those extracted from plasma [29] allow to envision validation procedures for the different contexts of use, entailing preclinical stages, or screening or case finding purposes, like in oncology. The present effort is still limited to the diagnosis of patients with objective clinical impairment in specialistic settings (memory clinics); however, validation studies relative to other contexts of use, which need to be assessed with specific reference to such different contexts, are increasingly conceivable. Future efforts may import methodology and validation status of Phases 1–2 of the current SBR as such, since the methodology of diagnostic studies assessing the performance of new assays is standardized whatever the contexts and adapt the framework for consistent data generation and assessment from Phase 3. On the whole, these future advances will further develop the research of AD diagnostic biomarkers consistently with the oncological model and in the direction of precision medicine.
